# AF-DETR: Transformer-Based Object Detection for Precise Atrial Fibrillation Beat Localization in ECG

**DOI:** 10.3390/bioengineering12101104

**Published:** 2025-10-14

**Authors:** Peng Wang, Junxian Song, Pang Wu, Zhenfeng Li, Xianxiang Chen, Lidong Du, Zhen Fang

**Affiliations:** 1 Aerospace Information Research Institute, Chinese Academy of Sciences, Beijing 100190, China; wupang@aircas.ac.cn (P.W.); lizhenfeng@aircas.ac.cn (Z.L.); chenxx@aircas.ac.cn (X.C.); lddu@mail.ie.ac.cn (L.D.); 2 Department of Cardiology, Peking University People’s Hospital, Beijing 100044, China; sjx221@163.com; 3 School of Electronic, Electrical and Communication Engineering, University of Chinese Academy of Sciences, Beijing 101408, China; 4 Personalized Management of Chronic Respiratory Disease, Chinese Academy of Medical Sciences, Beijing 100190, China

**Keywords:** atrial fibrillation, deep learning, electrocardiogram, object detection, cross-database validation

## Abstract

Atrial fibrillation (AF) detection in electrocardiograms (ECG) remains challenging, particularly at the heartbeat level. Traditional deep learning methods typically classify ECG segments as a whole, limiting their ability to detect AF at the granularity of individual heartbeats. This paper presents AF-DETR, a novel transformer-based object detection model for precise AF heartbeat localization and classification. AF-DETR incorporates a CNN backbone and a transformer encoder–decoder architecture, where 2D bounding boxes are used to represent heartbeat positions. Through iterative refinement of these bounding boxes, the model improves both localization and classification accuracy. To further enhance performance, we introduce contrastive denoising training, which accelerates convergence and prevents redundant heartbeat predictions. We evaluate AF-DETR on five publicly available ECG datasets (CPSC2021, AFDB, LTAFDB, MITDB, NSRDB), achieving state-of-the-art performance with F1-scores of 96.77%, 96.20%, 90.55%, and 99.87% for heartbeat-level classification, and segment-level accuracies of 98.27%, 97.55%, 97.30%, and 99.99%, respectively. These results demonstrate the effectiveness of AF-DETR in accurately detecting AF heartbeats and its strong generalization capability across diverse ECG datasets.

## 1. Introduction

Atrial fibrillation (AF) is the most common cardiac arrhythmia [1], with its global incidence and prevalence on the rise [2]. The presence of AF promotes thrombus formation, which may lead to stroke [3] and systemic embolism [4], and increases the risk of other cardiovascular diseases such as heart failure [3], myocardial infarction [5], and sudden cardiac death [6]. Currently, ECG signals serve as the gold standard for clinical AF diagnosis [7]. On an ECG, AF is characterized by irregular ventricular rhythms, absence of P waves, and presence of F waves [8]. According to guidelines, AF episodes lasting at least 30 s on ECG can be diagnosed as clinical AF [9]. AF is inherently progressive, meaning that it tends to worsen over time without intervention [10]. Therefore, early diagnosis of AF is crucial for timely intervention [11].

However, due to the often asymptomatic [12] and paroxysmal nature of AF, clinical screening for AF remains challenging. While palpitations, dyspnea, fatigue, and chest discomfort are common symptoms, a substantial proportion of AF patients remain asymptomatic, posing major challenges for timely detection and management. Recent evidence indicates that approximately 27% of AF patients are asymptomatic, with estimates ranging from 20% to 50% across populations [13]. Importantly, asymptomatic AF carries a risk of thromboembolic and cardiovascular events comparable to, or even higher than, symptomatic AF, largely due to delayed diagnosis and treatment. Moreover, asymptomatic status has been associated with specific clinical profiles—including male sex, diabetes mellitus, chronic kidney disease, and prior stroke or transient ischemic attack—that may predispose individuals to unrecognized AF episodes. Relying solely on infrequent and short-term rapid ECG assessments (30 s) for detecting AF may lead to underdiagnosis. It is further emphasized that patients at higher risk of AF prediction require long-term ECG monitoring [14] to increase the chances of early AF detection. In addition, there is still a need to rely on medical experts for interpreting ECG data. Manual visual inspection of ECG recordings is also a highly time-consuming and error-prone task. This significantly reduces the chances of timely detection of AF. Therefore, reliable automated methods must be developed for analyzing and interpreting long-term ECG recordings, particularly for asymptomatic individuals. Automated beat-level AF localization algorithms could facilitate earlier detection and personalized intervention, helping to prevent progression and reduce the burden of AF-related complications.

In recent years, machine learning (ML) and deep learning (DL) have been extensively developed in the field of AF detection [15]. Machine learning methods focus on classifying ECG segments using manually extracted features obtained through feature extraction and feature selection, with generally low computational costs in training and testing [16]. It is worth noting that the process of developing effective handcrafted features and finding the best-performing feature combinations is time-consuming and labor-intensive, heavily relying on manual operations. In the majority of ML-based AF detection approaches, QRS wave detection is a mandatory initial step. The performance limitations of QRS detectors and the presence of noise can affect the performance of the final ML model. Furthermore, there are significant differences in electrocardiographic signals among different patients, making it challenging to find effective ECG morphological features. Therefore, machine learning methods based on handcrafted features alone are insufficient for accurate AF detection. More and more studies are employing DL methods to achieve more accurate prediction results, albeit potentially requiring higher computational costs. Most DL-based methods utilize end-to-end frameworks with raw data as input to automatically extract features, allowing the model to learn feature embeddings most suitable for specific classification tasks. Deep learning demonstrates tremendous potential in automatically detecting and classifying arrhythmias from electrocardiographic signals and exhibits proficiency at a level comparable to that of medical experts in classifying various arrhythmias.

However, there are still some overlooked issues in existing methods that require further research. Firstly, the majority of existing AF detection methods generate a single prediction label for an ECG segment without providing beat-level prediction labels. An ECG segment may contain both AF and non-AF parts, leading to ambiguity in the feature space and misleading the model to embed them into overlapping feature spaces. Secondly, AF beat detection in long-term ECG recordings remains a challenge. The research on beat-level AF detection is significantly less explored and its performance needs improvement. Existing AF detection models expect isolated heartbeat signals as input for beat-level detection. Although shorter input segment lengths can achieve more fine-grained detection results, shorter ECG segments mean fewer modeled heartbeat dependencies, which often lead to poorer detection performance. Additionally, AF detection methods that use a single heartbeat signal or RRI sequence as input still require explicit heartbeat localization. Traditional threshold-based QRS detection algorithms still have limitations in various arrhythmia scenarios, and erroneous localization results may further restrict the detection capabilities.

To address these issues, this paper models the AF detection task as an object detection problem to achieve beat-level prediction. An AF object detection model takes fixed-length ECG segments as input and outputs classification results for AF beats along with position predictions. By capturing heartbeat dependencies, the model can enhance the classification performance of individual beats. Considering all beat classification results collectively also allows for segment-level classification predictions. The DETR architecture [17] has achieved significant success in the field of image object detection. Inspired by this, we introduce the DETR architecture for object detection in 1D ECG signals.

To be specific, this paper presents a novel AF object detection model named AF- DETR, which consists of a CNN feature extraction backbone, a transformer encoder, a dual-query transformer decoder, and several prediction heads. The AF-DETR model utilizes the CNN backbone to extract rhythm and morphology information from ECG signals and feeds a compact ECG feature representation into the transformer encoder to capture dependencies between heartbeats. The refined encoder ECG features are further decoded by the decoder to predict heartbeat positions and categories. In the decoder, decoder queries are divided into content and positional parts, and position queries are designed using 2D bounding boxes which represent heartbeat positions. Through iterative updates, the decoder progressively corrects the bounding boxes and captures crucial classification information, ultimately achieving accurate heartbeat localization and classification predictions. Additionally, a denoising training method is introduced during the training process to stabilize bipartite matching and accelerate model convergence. Positive and negative noise samples are constructed from the same ground truth to help the model avoid duplicate outputs of the same target. Cross-dataset testing is conducted using five datasets to evaluate beat-level localization and classification performance as well as segment-level classification performance, demonstrating the strong AF detection capabilities of the model. The contributions of this paper are as follows:

(1) A novel AF-DETR model with a CNN-Transformer architecture is proposed to model the AF detection as an object detection problem, thereby achieving localization and classification of AF heartbeats.
(2) Deriving decoder positional queries from 2D bounding boxes representing heartbeat positions, enabling the decoder to iteratively update bounding boxes layer by layer, thus accelerating model convergence and effectively localizing AF heartbeats.
(3) Denoising training is introduced to stabilize bipartite matching. Concretely, a contrastive denoising mechanism is conducted to help the model avoid duplicate detections of the same heartbeat by adding positive and negative noise samples.
(4) The proposed AF-DETR model achieves accurate detection of AF heartbeats in cross-dataset testing on four external datasets and demonstrates excellent generalization performance. Additionally, it performs comparably to existing methods in segment-level classification performance.

The remaining parts of this study are arranged as follows. Section 2 introduces the related work. Section 3 outlines the methods used in this study. Section 4 presents the datasets, experiments and results. Section 5 discusses the proposed method. Section 6 summarizes this study.

## 2. Related Work

The AF detection approaches can be roughly categorized into three types: methods based on ventricular rhythm information, methods based on atrial morphology information, and methods based on overall ECG information.

Methods based on ventricular rhythm typically involve extracting RR interval sequences and computing various handcrafted features, including multiple time-domain and frequency-domain features [18], various entropy features [19,20,21], Poincare plots [22,23], and Lorenz plots [24], to construct machine learning models such as support vector machine (SVM) [25,26,27], decision tree [28], k-nearest neighbor (KNN) [29], and random forest (RF) [30,31,32]. A few studies have constructed DL-based models with RR interval sequences as input [33,34]. Due to the good anti-interference performance of R-peak detection, methods based on irregular ventricular rhythm information generally exhibit good generalization performance. However, they are difficult to implement in short time series, and the irregular behavior of AF overlaps with other arrhythmias [35], leading to false positive detection in cases with frequent premature beats. Additionally, in some cases, the RR intervals of AF can also be regular, further limiting the effectiveness of rhythm-based methods.

Methods based on atrial morphology typically involve extracting P-wave/F-wave morphological features to determine the disappearance of P-waves and the appearance of F-waves [36,37]. To extract features from atrial activity, some studies isolate F- waves by eliminating the QRS complex from the ECG signal [38]. However, due to the small amplitude of P-waves and their susceptibility to various types of noise [39], the performance of AF detection algorithms based on atrial morphology information is not very robust. Therefore, the morphology-based methods are often used in conjunction with rhythm-based methods [40,41] to further improve the accuracy of AF detection.

Methods based on overall ECG information do not explicitly extract ventricular rhythm information or atrial morphology information but instead employ deep neural networks to achieve end-to-end AF detection. CNN backbones are commonly used for automatic feature extraction from ECG signals [42,43]. Some studies further refine the features extracted by CNN backbones using LSTM [44,45,46,47], GRU [48], and transformer encoders [49].

Traditional AF detection methods typically generate segment-level AF detection results. When finer-grained prediction labels are desired, reducing the length of input segments is a common approach, but this reduces the available heartbeat dependencies, leading to a sharp decrease in classification performance. This paper aims to achieve beat-level AF detection without reducing the length of input segments, using an object detection approach.

## 3. Method

### 3.1. Problem Definition

Beat-level atrial fibrillation detection can be expressed as an object detection task to predict the type and location of the target heartbeat. The AF-DETR architecture models object detection as a set prediction problem that directly infers N predictions. The fixed size N is set to be significantly larger than the number of objects in the sample, and an additional class label Non-obj is set to indicate cases where there is no object in the predicted bounding box.

A normalized ECG segment with a sampling frequency of $f_{s}$ and a duration of t seconds is used as a sample, which can be defined as $x_{ecg}\in R^{L_{0}\times 1}$, where the sample length is $L_{0}=t\times f_{s}$. The ground truth set of objects of the ECG sample is expressed as $y={\left\{y_{i}\right\}}_{i=1}^{M}$ and each element $y_{i}$ can be represented as $y_{i}=(c_{i},\;{\;b}_{i})$, where $c_{i}$ is the class label, $b_{i}=(x_{i},\;{\;w}_{i})$ is the ground truth box, $x_{i}\in \left[0,\;1\right]$ is the central coordinate, and $w_{i}\in \left[0,\;1\right]$ is the width relative to the sample length.

The AF-DETR model mainly consists of a backbone, a transformer encoder, and a dual-query transformer decoder. Samples are fed into the CNN-based backbone to extract a compact ECG feature representation. Then, the CNN features are refined by combining positional encoding and the encoder. The encoded ECG features are decoded to N objects in parallel by transformer encoder. The output of the final layer of the decoder generates the prediction label and the 2D prediction box, respectively, through the classification prediction head and the bounding box prediction head.

Since the predicted objects are not directly matched with the ground truth objects, the loss is calculated according to the optimal bipartite matching between the ground truth objects and the predicted objects during training. The AF-DETR model directly makes N predictions based on the input sample, and each prediction corresponds to only one GT object or Non-obj. To match the prediction set, the ground truth set y is considered as a set of size N filled with ∅ (Non-obj). The Hungarian algorithm can be used to efficiently compute an optimal bipartite matching between these two sets from the cost matrix of objects, which means to find a permutation of N elements $\sigma \in S_{N}$ with the lowest cost:

(1) $$\hat{\sigma }=\underset{\sigma \in S_{N}}{argmin}\sum_{i}^{N}\;L_{\mathrm{match}\;}\left(y_{i},{\hat{y}}_{\sigma (i)}\right)$$

where $L_{\mathrm{match}\;}\left(y_{i},{\hat{y}}_{\sigma (i)}\right)$ is a pair-wise matching cost between the ground truth $y_{i}$ and the prediction ${\hat{y}}_{\sigma (i)}$. The matching cost considers the location cost and the classification cost, respectively. For the prediction ${\hat{y}}_{\sigma (i)}$, the predicted box is defined as ${\hat{b}}_{\sigma (i)}$, and the prediction probability of $c_{i}$ class is defined as ${\hat{p}}_{\sigma (i)}$. The negative prediction probability is used as the classification cost, and the location cost is considered using the negative 1D generalized intersection over union (GIoU) and the *L*1 norm of the bounding boxes. The 1D GIoU and *L*1 norms for ground truth and prediction boxes can be calculated as:

(2) $$GIoU\left(b_{i},{\hat{b}}_{\sigma (i)}\right)=\frac{\left|b_{i}\cap {\hat{b}}_{\sigma (i)}\right|}{\left|b_{i}\cup {\hat{b}}_{\sigma (i)}\right|}-\frac{A\left(b_{i},{\hat{b}}_{\sigma (i)}\right)-\left|b_{i}\cup {\hat{b}}_{\sigma (i)}\right|}{A\left(b_{i},{\hat{b}}_{\sigma (i)}\right)}$$

(3) $$L1\left(b_{i},{\hat{b}}_{\sigma (i)}\right)={\left\|b_{i}-{\hat{b}}_{\sigma (i)}\right\|}_{1}$$

The symbol $\left|\cdot \right|$ refers to the length of the box, and A means the length of the smallest interval that covers both bounding boxes. Then, the matching cost adjusted by the hyperparameters $\varphi_{cls},\;\varphi_{giou}$ and $\varphi_{L1}$ can be expressed as:

(4) $$L_{\mathrm{matc}\;}\left(y_{i},{\hat{y}}_{\sigma (i)}\right)=-1_{\left\{c_{i}\neq \emptyset \right\}}\varphi_{cls}{\hat{p}}_{\sigma (i)}\left(c_{i}\right)-1_{\left\{c_{i}\neq \emptyset \right\}}\varphi_{giou}GIoU+1_{\left\{c_{i}\neq \emptyset \right\}}\varphi_{L1}L1$$

For the loss function, it is also necessary to consider both classification loss and positioning loss, and the total loss can be expressed as:

(5) $$L_{\mathrm{total}\;}(y,\hat{y})=\sum_{i=1}^{N}\;\left[\lambda_{cls}L_{cls}\left(y_{i},{\hat{y}}_{\hat{\sigma }(i)}\right)+I_{\left\{c_{i}\neq \emptyset \right\}}L_{box}\left(y_{i},{\hat{y}}_{\hat{\sigma }(i)}\right)\right]$$

where $\lambda_{cls}$ is the classification loss coefficient. The classification loss function adopts weighted multi-classification cross entropy loss to alleviate class imbalance, which can be expressed as:

(6) $$L_{cls}\left(y_{i},{\hat{y}}_{\hat{\sigma }(i)}\right)=-\alpha_{c_{i}}log{\hat{p}}_{\hat{\sigma }(i)}\left(c_{i}\right)$$

where a typical value $\alpha_{c_{i}}=0.1$ is used when $c_{i}=\emptyset$, and $\alpha_{c_{i}}=1$ is used in other cases. In addition, a linear combination of *L*1 loss and 1D GIoU loss is used as the positioning loss to improve the positioning accuracy. It is calculated as:

(7) $$L_{box}\left(y_{i},{\hat{y}}_{\hat{\sigma }(i)}\right)=\lambda_{giou}\left(1-GIoU(b_{i},{\hat{b}}_{\hat{\sigma }(i)})\right)+\lambda_{L1}{\left\|b_{i}-{\hat{b}}_{\hat{\sigma }(i)}\right\|}_{1}$$

where $\lambda_{giou}$ and $\lambda_{L1}$ are the hyperparameters for adjusting the loss weight.

### 3.2. CNN Backbone

**Figure 1:**
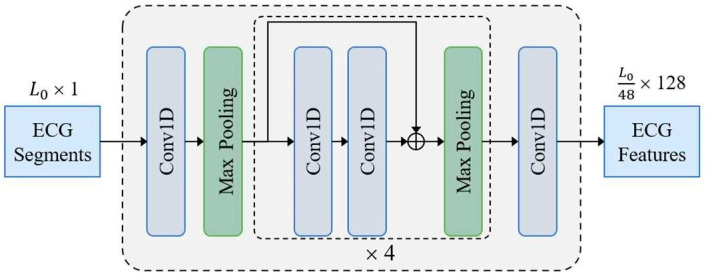
Structure of the CNN Backbone in the AF-DETR.

The CNN backbone constructed with 1D residual blocks is shown in Figure 1. The input sample $x_{ecg}\in R^{L_{0}\times 1}$ is fed to a 1D convolution layer and four residual blocks to acquire a compact ECG feature representation. Finally, the number of feature channels is adjusted to *D* = 128 through a 1D convolution layer with kernel size of 1. Except the last convolutional layer, all convolutional layers use a kernel size of 3 followed by a batch normalization layer and a ReLU activation layer. Each residual block contains two convolution layers and doubles the number of channels in the first convolution layer. In addition to setting the first max pooling layer with stride size of 3 and kernel size of 3, all max pooling layers have a stride size of 2 and a kernel size of 2. The ECG features extracted by the CNN backbone are fed to transformer for final predictions.

### 3.3. Transformer Encoder

**Figure 2:**
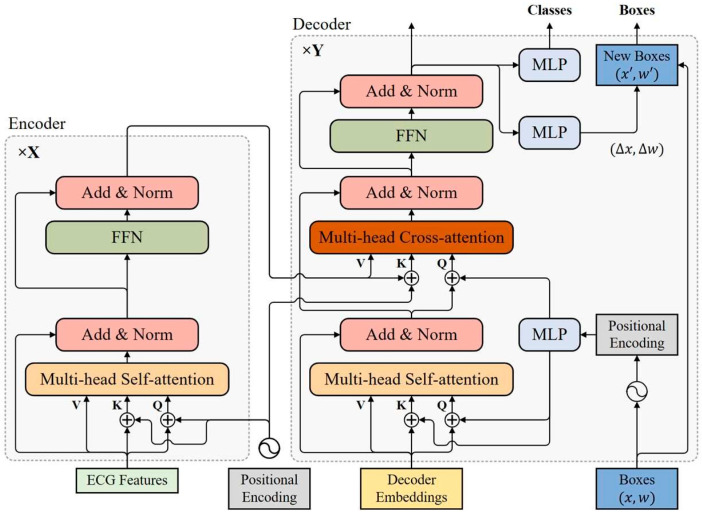
Structure of the transformer in the AF-DETR.

The transformer encoder consists of multiple encoder layers with the same structure, all of which are capable of encoding ECG features at the same resolution as the input. As shown in Figure 2, each encoder layer has a standard architecture consisting of an attention module and a feed forward network (FFN) with a residual connection around them, and followed by a layer normalization module. The feed forward network consists of two fully connected layers, which are connected using the ReLU activation layer.

#### 3.3.1. Positional Encoding

Because of the permutation invariance of transformer architecture, fixed positional encoding is added to the input of each attention module to capture positional dependencies, which helps complement the location representation capabilities of model. The sinusoidal function is used to construct a positional encoding vector on the same scale. The positional encoding function PE: $R\to R^{D}$, which maps position x to a D-dimensional sinusoidal embedding, is defined as:

(8) $$PE(x{)}_{2i}=sin\left(\frac{x}{T^{2i/D}}\right)$$

(9) $$PE(x{)}_{2i+1}=cos\left(\frac{x}{T^{2i/D}}\right)$$

where symbol x in the encoder refers to the position of the encoding vector in the time dimension. The subscripts 2i and 2i+1 represent the index in the encoding vector, The parameter T is a hand-designed temperature, and the typical value used in the encoder is T=10,000.

#### 3.3.2. Multi-Head Self-Attention Module

In each encoding layer, ECG features are combined with positional encoding to generate a query Q, a key K, and a value V, respectively, as inputs to the self-attention module. In the self-attention module, the query $Q\in R^{L\times D}$, key $K\in R^{L\times D}$, and value $V\in R^{L\times D}$ are linearly projected H times to obtain H groups $Q_{i}=QW_{i}^{Q}\in R^{L\times d_{q}}$, $K_{i}=KW_{i}^{K}\in R^{L\times d_{k}}$, and $V_{i}=VW_{i}^{V}\in R^{L\times d_{v}}$, respectively, preparing for multi-head self-attention. A typical setting is $d_{q}=d_{k}=d_{v}=D/h$ and the weight matrices $W_{i}^{Q}\in R^{D\times d_{q}},\;W_{i}^{K}\in R^{D\times d_{k}}\;\mathrm{and}\;W_{i}^{V}\in R^{D\times d_{v}}$ are learnable parameters of linear projection layers. The scaled dot-product attention function for each head can be expressed as:

(10) $$Attention\left(Q_{i},K_{i},V_{i}\right)=Softmax\left(\frac{Q_{i}K_{i}^{T}}{\sqrt{d_{k}}}\right)V_{i}$$

The output of all heads is concatenated in the feature dimension and fed to a linear layer with a weight parameter $W^{o}\in R^{D\times D}$ to produce the final output, which can be expressed as:

(11) $$\mathrm{MultiHeadAttn}(Q,K,V)=Cat(A_{1},A_{2},\cdots ,A_{h})W^{o}$$

where $A_{i}=Attention\left(Q_{i},K_{i},V_{i}\right)\in R^{L\times d_{k}}$ is the output of the *i*-th head. The symbol Cat represents the concatenation function.

### 3.4. Transformer Decoder

The transformer decoder uses multiple decoder layers to decode N targets in parallel. Each decoding layer uses multi-head self-attention modules to update queries and cross-attention modules to probe the objects based on the similarity of queries and keys. The decoder updates the query layer by layer and continuously approximates the target ground-truth objects. Considering that the keys in the cross-attention module contain both the content part (encoded ECG features) and the location part (positional embedding), a dual-query mode consisting of content queries and positional queries is introduced, where the initial content query uses decoder embeddings.

#### 3.4.1. Positional Query

In order to make the positional similarity of queries and keys in the cross-attention module more consistent, the bounding boxes are directly learned in each decoder layer and positional queries are derived from these bounding boxes with sinusoidal positional encoding functions. The *q*-th box of N bounding boxes is defined as $b_{q}={(b}_{qx},\;b_{qw})$, where $b_{qx}\in R$ and $b_{qw}\in R$. The corresponding content query and positional query are represented as $C_{q}\in R^{D}$ and $P_{q}\in R^{D}$, respectively, where D is the dimension of decoder queries. The positional query $P_{q}$ derived from the given bounding box $b_{q}$ is:

(12) $$P_{q}=MLP\left(PE\left(b_{q}\right)\right)=MLP\left(Cat\left(PE\left(b_{qx}\right),PE\left(b_{qw}\right)\right)\right)$$

where the symbol PE represents the positional encoding function $PE:\;R\to R^{D}$ that maps a float number to a *D*-dimensional sinusoidal embedding. A hyperparameter setting T = 20 is used in decoder. The symbol MLP represents a multi-layer perceptron $MLP:R^{2D}\to R^{D}$, which projects a *2D* vector into *D*-dimensional vector. It includes two fully connected layers and its parameters are shared between all decoder layers.

For the initial queries of the first decoding layer, the decoder embeddings and initial bounding boxes need to be prepared, respectively. Here, the decoder embeddings are designed to be learnable embeddings, while the initial bounding boxes adopt a fixed box width and a uniformly distributed center point.

#### 3.4.2. Self-Attention Module of Decoder

The self-attention module in the decoder is used to query updating the cross-attention module. All queries, keys, and values use the same content item $C_{q}$, while queries and keys contain an additional positional item $P_{q}$. The input of the self-attention module in the *d*-th decoding layer can be expressed as:

(13) $$Q_{q}^{d}=C_{q}^{d}+P_{q}^{d}$$

(14) $$K_{q}^{d}=C_{q}^{d}+P_{q}^{d}$$

(15) $$V_{q}^{d}=C_{q}^{d}$$

#### 3.4.3. Cross-Attention Module of Decoder

The cross-attention module in the decoder also uses a multi-head attention mechanism. In the cross-attention module, positional and content information are combined as object queries to extract object information from the encoded ECG features. The input of the cross-attention module in the d-th decoder layer can be expressed as:

(16) $$Q_{q}^{d}=LN\left(C_{q}^{d}+S_{q}^{d}\right)+P_{q}^{d}$$

(17) $$K_{x}^{d}=F_{x}+PE(x)$$

(18) $$V_{x}=F_{x}$$

where $F_{x}$ is the feature vector at position x in the encoded ECG feature, *S*$S_{q}^{d}$ is the output of the self-attention module in the decoding layer *d*, and *LN* represents the layer normalization function.

#### 3.4.4. Iterative Bounding Box Updating

The pattern of deriving positional queries from bounding boxes makes it possible to update bounding boxes layer by layer. With the exception of the first decoding layer, each decoder layer updates positional queries based on the updated bounding boxes of the previous layer. Each layer predicts the relative offset of the bounding box $\left(\Delta b_{qx},\Delta b_{qw}\right)$ for the next layer through the unshared bounding box prediction head $MLP:R^{D}\to R^{2}$, which helps to further reduce optimization difficulty. All bounding box prediction heads contains three fully connected layers. Given a bounding box ${\hat{b}}_{q}^{d-1}$ provided by the (*d* − 1)-th decoder layer, the d-th decoder layer updates the bounding box as:

(19) $$b_{q}^{d}=\left\{\sigma \left(\Delta b_{qx}^{d}+\sigma^{-1}\left(b_{qx}^{d-1}\right)\right),\sigma \left(\Delta b_{qw}^{d}+\sigma^{-1}\left(b_{qw}^{d-1}\right)\right)\right\}$$

where the symbols σ and $\sigma^{-1}$ represent the sigmoid function and the inverse sigmoid function, respectively.

#### 3.4.5. Predicted Output

In addition to using the updated bounding boxes of each decoder layer as its box prediction, the normalized output of each decoder layer is fed to an unshared classification prediction head $MLP:R^{D}\to R^{3}$ to make classification predictions. All classification prediction heads consist of two fully connected layers. Hungarian matching is applied to predicted results of each decoder layer against the ground truth objects, and auxiliary loss of each decoder layer is built to update parameters, which helps speed up model convergence. For stable training, the bounding box prediction head and classification prediction head of each decoder layer use only its auxiliary loss for parameter updating. The predictions of the last decoder layer are the predictions of the whole model. When inference is performed, predictions with a Non-obj label can be omitted, and other valid predictions need to be retained.

### 3.5. Denoising Training

In order to mitigate the instability caused by Hungarian matching, denoising (DN) training is introduced, which is able to reconstruct the object from the noised ground truth. Therefore, random noise is added to the bounding box and class label of each ground truth object to construct additional noisy query. Similar to the ordinary query, the noise query is also composed of content query and positional query. The noised labels generate the initial content queries of the decoder, and the noised bounding boxes generates the initial positional queries. Noised bounding boxes and noised labels are constructed as follows:

(1) Noised bounding boxes: The method of center shifting is adopted for adding noise to bounding boxes. Random noise Δx satisfying $|\Delta x|<\frac{\lambda w}{2}$ is added in the center point of the box, so that the center of the shifted box is still in the original box. The random noise is controlled by the noise scale parameter λ. Contrastive denoising (CDN) training is further considered to help the model avoid duplicate predictions of the same object. Contrast denoising training generates positive and negative queries by setting two different parameters $\lambda_{1}$ and $\lambda_{2}$. A positive query which satisfies $|\Delta x|<\frac{\lambda_{1}w}{2}$ is expected to reconstruct its corresponding ground truth box. Negative queries satisfy $\frac{\lambda_{1}w}{2}\leq |\Delta x|\leq \frac{\lambda_{2}w}{2}$, and they are expected to be predicted as Non-obj.
(2) Noised labels: Noised labels are acquired by randomly flipping some ground truth labels to other labels, and the proportion of label flipping is controlled the hyperparameter γ. For the noise query, label embedding generated by the noised label is used as the content part.

In fact, multiple groups of noisy queries can be constructed. It is important to note that despite the addition of noise, the noise query contains information about the true object. In order to prevent information leakage, when the self-attention module of the decoder calculates the attention weight matrix, it is necessary to use attention mask isolation between ordinary queries and multi-group noise queries. The predictions corresponding to noise queries can bypass bipartite matching and directly calculate its reconstruction loss with the corresponding ground truth objects. The reconstruction loss of noise queries is similar to that of ordinary queries. Noise queries are only added to the decoder during training and removed during inference.

## 4. Experiments and Results

### 4.1. Datasets

In this work, five publicly available datasets from Physionet [50] were used. They all have manually corrected heartbeat positional annotations and label annotations. The datasets are described as follows:

(1) MIT-BIH Atrial Fibrillation Database (AFDB) [51]: The AFDB includes long-term ECG recordings from 25 human subjects with AF (mostly paroxysmal), each lasting 10 h. Each record contains two ECG signals, sampled at a rate of 250 samples per second.
(2) The 4th China Physiological Signal Challenge 2021 (CPSC2021) [52]: The database provides two training sets, which together comprise 1406 records extracted from dynamic ECG recordings of 49 AF patients (23 paroxysmal AF patients) and 56 non-AF patients (including other abnormalities and normal rhythms). Each ECG recording provides two channel signals of leads I and II at 200 Hz.
(3) Long Term AF Database (LTAFDB) [53]: The LTAFDB consists of long-term ECG recordings from 84 subjects with paroxysmal or persistent AF. Each record is digitized at 128 Hz and has varying durations, typically ranging from 24 to 25 h.
(4) MIT-BIH Arrhythmia Database (MITDB) [54]: The dataset comprises 48 half-hour dual-channel dynamic ECG recordings, digitized at 360 Hz. Each record is independently annotated by two or more cardiac experts. The ECG signals primarily consist of lead II and V1.
(5) MIT-BIH Normal Sinus Rhythm Database (NSRDB) [50]: The dataset provides long-term ECG recordings from 18 subjects without obvious arrhythmias. Each record contains two ECG lead signals sampled at a frequency of 128 Hz, and corresponding beat annotation files are available.

Table 1 summarizes detailed information about the five open-source datasets. All records in the datasets provide dual-channel dynamic ECG signals. When lead information is available, channels containing leads I and II are prioritized. If this information is not available, channels similar to lead II are selected from the two channels for analysis. Only one lead was used for model input. When two leads were available, Lead II was prioritized for both training and testing because of its stable morphology and common use in single-lead wearables. It should be noted that the record “07162” in the AFDB dataset was excluded from the analysis in this paper due to a large number of beat annotation errors.

The dynamic ECG recordings were resampled to 128 Hz to ensure a consistent sampling rate across all datasets. A bandpass Butterworth filter with a filter order of 5 and passband frequency range of 0.5 to 40 Hz was applied to remove baseline drift and high-frequency noise from the ECG signals. Each record was segmented into non-overlapping 30 s ECG segments for analysis, and each ECG segment was normalized using z-score normalization. To remove segments with severe noise, a signal quality index based on R-peak detection (bSQI) was used to assess the signal quality, and the ECG segments with bSQI < 0.8 were excluded from the analysis. In addition, to increase sample diversity, the number of ECG fragments was amplified using a data augmentation method of vertical flipping [55]. This operation mimics the physiological polarity difference between lead I and lead II configurations across datasets, allowing the model to learn polarity-invariant morphological features rather than memorizing lead-specific signal directions. Since AF detection primarily relies on rhythm irregularity and the presence of fibrillatory activity instead of absolute waveform polarity, this augmentation does not alter the underlying rhythm pattern. To further verify this, we conducted an ablation study comparing models trained with and without polarity inversion. According to the annotations of the datasets, each heartbeat in each segment was labeled as AF/AFL (positive) or Non-AF (negative); AF and AFL were included in the positive class, whereas all other rhythms were labeled as Non-AF. Table 2 summarizes the details of the segmentation for each dataset.

### 4.2. Experimental Setup

#### 4.2.1. Experimental Environment

Under the hardware configuration of Intel(R) Xeon(R) E5-2640 CPU (Beijing, China) and NVIDIA GeForce RTX 3090 GPU (Beijing, China), the training and testing of the proposed AF-DETR model were implemented using Python 3.9.15 with PyTorch 1.12.1 and CUDA 11.6.

#### 4.2.2. Training Setup

Each bounding box of heartbeat consists of a center position and width. The R-peak positions are used as the center of the boxes and an empirical value of 400 ms is adopted as the width of the ground truth boxes and the initial bounding boxes of decoder. To evaluate the robustness of the empirical 400 ms width across different cardiac rhythms, we further conducted an ablation experiment on the heartbeat-box width. Four width strategies were compared:

(1) Fixed-300 ms, a narrow window approximating fast heart rates (~100 bpm);
(2) Fixed-400 ms, the default configuration used in the main experiments;
(3) Fixed-500 ms, a broader window approximating slow heart rates (~60 bpm) and wide-QRS morphologies;
(4) Adaptive-RR, in which the box width is dynamically defined as 0.5 × the local R-R interval estimated from adjacent beats.

**Figure 3:**
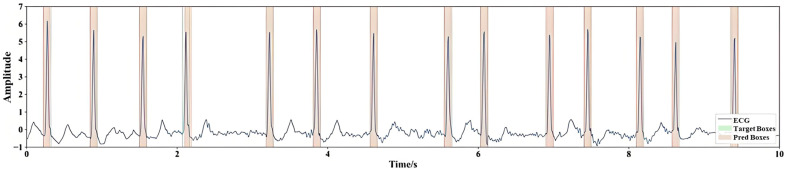
Visualization of ground truth boxes and predicted boxes.

During training, all other settings (optimizer, learning rate, epoch number, and dataset split) were identical. The matching between predicted and ground-truth boxes was still performed by the Hungarian algorithm to ensure one-to-one correspondence, thereby preventing collisions when boxes overlapped at high heart rates. The center position and width of each box are normalized to [0, 1]. Figure 3 visualizes the setup for the ground truth boxes and the desired effect of the predicted boxes. For the transformer part, we use 4 encoder layers and 4 decoder layers, with each feature length set to *D* = 128. The number of heads for multi-head attention is set to *H* = 8. During training, we use the adaptive moment estimation algorithm (Adam optimizer) to optimize the model. The hyperparameters involved in the network are manually fine-tuned. Finally, the cost coefficient for bipartite matching is determined as $\varphi_{cls}:\varphi_{L1}:\varphi_{giou}=2:5:2$, and the loss function coefficient is $\lambda_{cls}:\lambda_{L1}:\lambda_{\mathrm{giou}\;}=1:5:2$. The initial learning rate $\eta_{\mathrm{base}\;}$ is set to 0.0001, and the batch size is 64. The formula for the learning rate change with training epochs is:

(20) $$\eta =\eta_{\mathrm{base}\;}/{\left(1+10\times T_{\mathrm{cur}\;}/T_{max}\right)}^{2}$$

where $T_{\mathrm{cur}\;}$ represents the current training epoch, and $T_{max}$ represents the total number of training epochs. In this work, $T_{max}$ is set to 30.

#### 4.2.3. Testing Setup

In this work, we primarily conducted external independent testing, where different datasets were used for training and testing. This cross-dataset testing approach ensures that data segments from the same patients do not appear in both the training and validation sets, thereby better validating the generalization and robustness of model. We used the CPSC2021 dataset as the training set because it has the largest number of subjects, an adequate number of samples, a relatively balanced data distribution, and clear lead information, which helps evaluate the real performance of model. Other datasets were further used for external independent testing.

#### 4.2.4. Evaluation Metric

In this work, the overall performance of the model needs to be evaluated by separately assessing its localization performance and classification performance. Metrics such as Accuracy (*Acc*), Sensitivity (*Sen*), Precision (*Pre*), *F*1-score (*F*1), etc., can be used to measure the localization performance and classification performance. When using True Positives (*TP*), False Positives (*FP*), False Negatives (*FN*), and True Negatives (*TN*) to represent these metrics, they are defined as follows:

(21) $$Acc=\frac{TP+TN}{TP+TN+FP+FN}$$

(22) $$\mathrm{Pre}=\frac{TP}{TP+FP}$$

(23) $$\mathrm{Sen}=\frac{TP}{TP+FN}$$

(24) $$F1=2\times \frac{Pre\times Sen}{Pre+Sen}$$

### 4.3. Results

#### 4.3.1. Localization Performance

To evaluate the heartbeat detection performance of AF-DETR, the ground truth boxes were matched with predicted boxes that are not labeled as Non-obj. A prediction box is considered as a correct detection when its IoU with a ground truth box is greater than 0.5. Precision and sensitivity are used as metrics to evaluate the positioning performance. We follow these rules: (1) TP is defined as correct detections of ground truth boxes. (2) FP is defined as incorrect detections where no object exists, or where the position of an object is incorrectly detected. (3) FN is defined as the cases where ground truth boxes are not detected. In addition, the predicted boxes with the same prediction label and an IoU greater than 0.8 are merged to ensure correct statistics.

Cross-dataset validation was conducted to evaluate the localization performance of AF-DETR. It should be noted that all tests performed were external independent tests using the model trained on the CPSC2021 dataset. Table 3 shows the localization results of the AF-DETR model on four external datasets. In the localization task, AF-DETR achieved the highest precision (Pre 99.79%) and sensitivity (Sen 99.96%) on the NSRDB, indicating that AF-DETR can achieve nearly perfect heartbeat localization for sinus rhythm. The sensitivity on AFDB, LTAFDB, and MITDB was 99.21%, 99.09%, and 99.54%, respectively, indicating that this method can accurately detect heartbeats of various arrhythmias, including AF. Compared to sensitivity, the precision on AFDB, LTAFDB, and MITDB was generally lower, with the lowest precision observed on MITDB (Pre 98.39%), suggesting that frequent arrhythmias may lead to increased false detection. However, despite the slight decrease in performance, the model still demonstrates good overall localization accuracy.

In addition, we considered the localization accuracy of each correctly detected bounding box. The distance of the center points between the predicted and ground truth boxes was further analyzed, and the mean absolute error (MAE) was used to represent localization accuracy. The average absolute errors of the AF-DETR model on AFDB, LTAFDB, MITDB, and NSRDB were 13.71 ms, 12.29 ms, 10.66 ms, and 5.81 ms, respectively. Considering the signal sampling rate of 128 Hz, the average absolute errors in localization are all less than 2 sampling points, which is acceptable.

#### 4.3.2. Classification Performance

In order to better observe the classification performance of the AF-DETR model, the heartbeat-level classification performance and segment-level classification performance should be separately analyzed and reported.

##### Beat-Level Performance

For the heartbeat classification task, AF-DETR actually makes predictions of three classes: Non-AF, AF, and Non-obj. However, a large number of Non-obj predictions are not the intended objects. Although including the Non-obj predictions in the statistics may lead to an overall inflated classification performance, it does not reflect the actual classification performance. Therefore, only the classification performance of the Non-AF and AF classes is evaluated, and the average of the two class performance metrics is used to represent the overall classification performance. Building upon the aforementioned localization rules, the matching between predicted labels and true labels is further refined to evaluate the heartbeat-level classification performance of AF-DETR on external datasets. It is worth noting that once the localization detection is incorrect, the corresponding heartbeat classification prediction results are also incorrect, which imposes higher demands on the performance.

**Figure 4:**
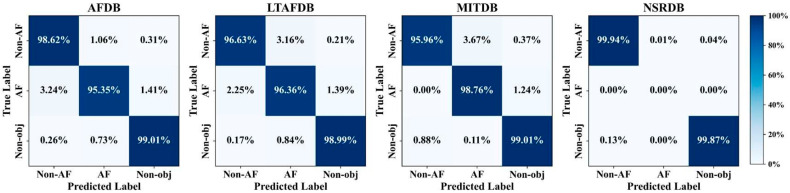
The confusion matrixes of beat-level classification results on the external datasets.

Figure 4 illustrates the confusion matrices on external datasets, while Table 4 presents the classification results of Non-AF and AF on external datasets. AF-DETR achieved F1-scores of 96.77% and 96.20% on AFDB and LTAFDB, respectively. Considering the relatively balanced data distribution in these two datasets, this indicates that AF- DETR effectively extracts neighboring heartbeat information to achieve robust AF heartbeat detection. On MITDB, 98.76% of AF heartbeats and 95.96% of Non-AF heartbeats were accurately localized and classified. Among Non-AF heartbeats, 3.67% were misclassified as AF, while there were almost no AF heartbeats misclassified as Non-AF. Furthermore, due to the extremely imbalanced data distribution in MITDB, the AF class exhibited a lower precision (Pre 73.02%). For the NSRDB dataset, which only contains Non-AF samples, only the classification performance of the Non-AF class is reported. Similar to the localization results, the best classification results (Pre 99.79%, Sen 99.94%, F1 99.87%) were achieved on NSRDB, where 99.99% of heartbeats were accurately classified among correctly localized heartbeats. Overall, these experimental results confirm the good heartbeat classification performance of AF-DETR method on external datasets.

##### Segment-Level Performance

Since the AF-DETR model is designed for heartbeat classification, AF-DETR needs to undergo inference and postprocessing to provide segment-level prediction labels. Here, the accuracy of heartbeat localization does not need to be considered. Instead, all AF and Non-AF heartbeat predictions from the model are adopted to assign segment-level prediction labels according to postprocessing rules. In this work, when the number of predicted AF heartbeats in an ECG segment exceeds 50%, the classification output label of that segment is determined to be AF; otherwise, it is predicted as Non-AF.

**Figure 5:**
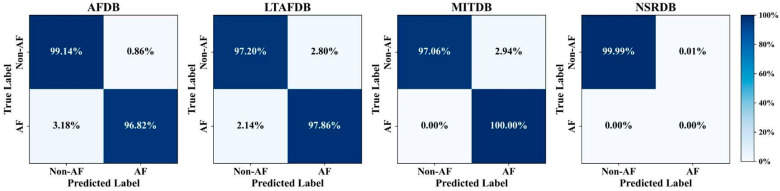
The confusion matrixes of segment-level classification results on the external datasets.

To further validate the generalization ability of AF-DETR, we conducted segment- level external independent testing. Similarly, the model trained on CPSC2021 was tested on other public datasets. The experimental results of external independent testing are summarized in Table 5, with the corresponding confusion matrix results shown in Figure 5. The AF-DETR model achieved the highest accuracy (98.24%) and F1-score (98.15%) of AF detection on the AFDB dataset, while on the LTAF dataset, the accuracy and F1-score were 97.55% and 97.54%, respectively. However, the AF-DETR model had the lowest accuracy (97.30%) and F1-score (92.31%) on the MITDB dataset, which is related to the imbalanced data distribution and the presence of a greater variety of arrhythmias. On the NSRDB dataset, due to the excellent performance of heartbeat classification, AF-DETR also achieved 99.99% accuracy in segment classification. Overall, the segment classification performance has been further improved based on the heartbeat classification performance, validating that the AF-DETR model can screen suspicious AF heartbeats and provide more reliable predictions for ECG records.

As the beat-level precision and segment-level precision on MITDB were lower than on other datasets, an extended rhythm-wise error and calibration analysis was conducted to identify the specific sources of false positives and to evaluate probability calibration (see Section 4.3.5).

To further examine the validity and clinical consistency of the post-processing rule used for segment-level labeling, an extended sensitivity and temporal continuity analysis was performed. In the default configuration, a 30 s segment was labeled as atrial fibrillation (AF) when more than 50% of its constituent beats were predicted as AF. Although this majority-vote rule has been widely employed in previous AF detection studies, it is a heuristic criterion rather than a clinical definition. From a clinical standpoint, an AF episode is defined by the persistence of fibrillatory activity for at least 30 s, irrespective of the proportion of AF beats within the interval.

To assess the robustness of this threshold, a threshold-sensitivity analysis was conducted by varying the AF-beat ratio parameter θ from 0.3 to 0.7 in increments of 0.1. For each θ, segment-level accuracy and F1-score were calculated under the CPSC2021→AFDB cross-dataset setting, with all other configurations kept constant. The aim was to determine whether the default 50% criterion introduces bias or whether the performance remains stable across a reasonable range of thresholds.

In addition, a time-consecutive criterion was introduced to better align the post-processing decision with clinical diagnostic standards. Under this criterion, a segment was labeled as AF only if the predicted AF beats formed a continuous time span lasting at least 30 s (corresponding to 3840 samples at 128 Hz). This formulation directly follows the clinical definition of an AF episode lasting ≥ 30 s and serves as a temporal-consistency verification of beat-level predictions.

The quantitative results of the two analyses are presented in Table 6 and Table 7.

The results demonstrate that AF-DETR maintains consistent segment-level accuracy and F1-scores under different threshold settings and time-based criteria. The performance difference between the two rules (<0.1%) indicates that the beat-level predictions exhibit strong temporal continuity, effectively satisfying the clinical requirement of ≥30 s of persistent AF activity. Hence, the 50% majority-vote criterion can be regarded as a practical and clinically consistent approximation of the time-based AF definition.

#### 4.3.3. Ablation and Robustness Studies

##### Ablation on Performance of AF-DETR

To identify factors contributing to the performance improvement of the model, we removed or modified certain components or methods of the model during the training process and tested them under the original experimental conditions. Here, AF-DETR is used as the baseline for comparison, and we sequentially studied the effects of components such as the CDN mechanism in the denoising training and auxiliary loss. Table 8 presents the ablation study results on the MITDB dataset regarding both heartbeat localization and classification.

The results of the ablation experiments indicate that the auxiliary loss is crucial for improving both heartbeat localization accuracy and classification performance. After removing auxiliary loss, there is a significant decrease in the precision and sensitivity of heartbeat localization, a substantial increase in mean absolute error, and a subsequent decline in heartbeat classification performance. In fact, the auxiliary loss can bring additional parameter updates to the classification prediction head and bounding box prediction head of each decoding layer, which helps the decoding layers extract the classification information faster and give more accurate bounding box updates, thus improving the model’s performance.

For the denoise training, the CDN mechanism is removed firstly. Although there was a slight improvement of 0.40% and 0.31% in the precision and sensitivity of heartbeat localization, respectively, there was a noticeable decrease in heartbeat classification performance. Moreover, after removing the denoising training mechanism entirely, both heartbeat localization and classification performance showed significant declines. We can infer that denoising training and the CDN mechanism have a significant positive impact on performance. The denoising component does not require bipartite matching, which avoids the instability caused by bipartite matching, thus accelerating model convergence and helping to improve object detection performance.

##### Ablation on Bounding-Box Width

Table 9 summarizes the influence of different heartbeat-box widths on localization and classification metrics using the CPSC2021→AFDB external-testing protocol. The results show that the proposed 400 ms setting already achieves near-optimal performance. The adaptive-RR strategy yields only marginal improvements (<0.1% in AF F1 and 0.3 ms in MAE), confirming that the fixed empirical width is sufficiently robust across different heart-rate ranges and QRS morphologies. Moreover, the matching algorithm effectively resolves potential overlaps between adjacent boxes at high heart rates, ensuring stable localization statistics.

##### Ablation on Polarity Inversion Augmentation

To assess the physiological plausibility and quantitative influence of the polarity inversion (vertical flipping) augmentation, we conducted an ablation experiment in which the AF-DETR model was retrained without vertical flipping, while keeping all other configurations identical. This analysis aimed to verify whether polarity inversion affects the visibility of P/F waves or alters rhythm recognition in single-lead ECGs.

Table 10 demonstrates that polarity inversion augmentation produces negligible changes (<0.05%) in AF F1, confirming that it does not distort rhythm characteristics or hinder the visibility of atrial fibrillatory waves. These results suggest that polarity inversion provides lead-polarity robustness—helping the model generalize to datasets recorded with opposite electrode configurations—without sacrificing physiological interpretability. In addition, polarity inversion yields a slightly higher IoU and lower MAE, indicating marginally improved heartbeat localization stability. This confirms that the augmentation is safe and effective for enhancing cross-dataset generalization, while preserving the clinical meaning of single-lead ECG morphology.

##### Lead-Swap Robustness Test

To evaluate robustness against lead variations, we conducted a lead-swap experiment on the CPSC2021 dataset, which provides paired Lead I and Lead II signals. The model was trained using Lead I signals and tested on Lead II (Lead-swap setting), while the default setting used Lead II for both training and testing. Table 11 shows the comparison. AF-DETR achieved almost identical localization and classification performance under the lead-swap configuration, with only a minor 0.36% drop in AF F1, indicating strong cross-lead generalization and confirming that the model can operate reliably on different single-lead configurations encountered in wearable ECGs.

#### 4.3.4. Cross-Database Generalization and Statistical Robustness

To verify that the observed performance is not specific to the CPSC2021 dataset characteristics, we performed symmetric cross-database experiments. AF-DETR was trained on CPSC2021 and tested on AFDB and MITDB (default setting), and vice versa. In addition, a joint training scenario combining CPSC and AFDB was evaluated. Each experiment was repeated three times with different random seeds (42, 3407, 9821), and mean ± 95% confidence intervals are reported in Table 12.

The results indicate that AF-DETR maintains high accuracy across different sampling rates and labeling protocols, with less than 0.7% variation in AF F1 across databases. This demonstrates that the model generalizes well to unseen datasets and that its performance is not idiosyncratic to CPSC2021. Furthermore, no patient overlap exists among the PhysioNet databases used, as each repository originates from independent clinical cohorts.

#### 4.3.5. Rhythm-Wise Error Analysis and Probability Calibration on MITDB

This subsection provides a detailed rhythm-wise error and calibration analysis corresponding to the classification results reported in the Beat-Level Performance Section, focusing on the MITDB dataset where lower precision was observed.

To identify the primary sources of false positives in the MIT-BIH Arrhythmia Database (MITDB), a rhythm-wise analysis was conducted, as shown in Table 13. MITDB contains 48 records with beat- and rhythm-level annotations, comprising 109,494 heartbeats from 47 subjects, and includes a broad distribution of ectopic and conduction abnormalities. False-positive (AF) beats produced by AF-DETR (trained on CPSC2021 using cross-entropy loss) were mapped to reference rhythm labels according to the official MITDB annotation files. Five confounding rhythm categories were considered: premature atrial contractions (PAC), premature ventricular contractions (PVC), bigeminy (atrial or ventricular), atrioventricular (AV) conduction block, and noise or motion artifacts.

PAC and PVC together accounted for 58% of all false positives, indicating that transient RR-interval irregularities and wide QRS morphologies constitute the major sources of misclassification. Bigeminal patterns contributed 18%, whereas AV block and noise segments accounted for the remainder.

To reduce the influence of these confounders, additional training was performed using focal loss (γ = 2, α = 0.75) and class-weighted cross-entropy (inverse-frequency weighting). All other training parameters were kept identical. Performance metrics were averaged over three independent runs (random seeds 42, 3407, 9821) with 95% confidence intervals. The results for beat-level AF detection on MITDB under the different loss functions is shown in Table 14.

The application of focal loss increased AF precision by 5.5% points and improved overall F1 by 1.5 percentage points compared with the default cross-entropy training. Analysis of rhythm-specific results indicated an 18% reduction in PAC-related false positives and a 13% reduction in PVC-related false positives, with minimal loss of recall.

Per-beat probability calibration was further examined using the expected calibration error (ECE) and Brier score. The focal-loss model achieved an ECE of 0.046 and a Brier score of 0.052, compared with 0.081 and 0.067 for the cross-entropy baseline, respectively, demonstrating improved alignment between predicted probabilities and empirical outcomes.

Overall, the analyses confirm that ectopic beats, particularly PAC and PVC, are the principal sources of false-positive AF detections on MITDB. Loss-reweighting strategies such as focal loss and calibrated class weighting effectively mitigate these errors and enhance model calibration. These results also imply that the inclusion of auxiliary tasks, such as ectopy or signal-quality classification, may further improve robustness in future developments.

#### 4.3.6. Comparison with Previous Works

For decades, automatic QRS wave detection has been a significant topic in ECG analysis, with numerous studies focusing on QRS detection algorithms. In this study, we attempted to compare the AF-DETR model with several QRS wave detection algorithms. The NeuroKit2 [56] toolkit was used to replicate these QRS wave detection algorithms [57,58,59,60], and they were tested on ECG segments segmented from the MITDB dataset. Unlike AF-DETR, these QRS wave detection algorithms used ECG segments that were not z-score normalized. When calculating performance metrics, we applied the same criteria used for AF-DETR to these other QRS wave detection algorithms. Using a default box width of 0.4 s, the predictions of these QRS wave detection algorithms were converted into prediction boxes, and then metrics such as TP, FP, FN, Pre, and Sen were calculated. Table 15 presents the comparison results between the AF-DETR model and other QRS wave detection algorithms. The comparison results indicate that the localization performance of the AF-DETR model is comparable to that of other algorithms. Additionally, the performance of AF-DETR is slightly better than that of the well-known Pan-Tompkins [57] algorithm. The proposed model demonstrates strong competitiveness with existing methods.

In addition to localization performance, we further attempted to compare the AF classification performance of AF-DETR with existing studies. However, few studies report cross-dataset evaluation results for AF object detection, which limits the number of papers available for comparison. Therefore, we included some studies on segment-level AF classification that underwent cross-dataset testing for comparison. Despite differences in preprocessing strategies and training settings among studies, we tested them using the same training sets and testing sets to supplement the results of AF-DETR. Table 16 presents the comparison results of the proposed method with existing works in terms of classification performance. From various metrics, it can be observed that the proposed model outperforms previous studies.

## 5. Discussion

### 5.1. Overview of the Proposed Method

In this work, an AF object detection model called AF-DETR is proposed to accurately localize AF heartbeats in single-lead ECG records. In the transformer decoder, decoder positional queries are constructed using 2D bounding boxes which represent the heartbeat positions, enabling iterative updates of the bounding boxes for precise heartbeat localization and classification prediction. Additionally, a denoising training method is introduced to stabilize bipartite matching and accelerate model convergence, while contrasting denoising mechanisms to avoid redundant predictions of the same heartbeat. Cross-dataset testing results demonstrate the excellent detection accuracy and generalization performance of the proposed method, making it applicable to various datasets without the need for parameter adjustments in practical applications.

### 5.2. Method Evaluation

The DETR architecture is introduced for object detection in 1D ECG signals. The research results suggest that the DETR architecture has the potential to improve the accuracy of physiological signal analysis, which could have a broader impact on the field of physiological signal diagnosis.

For AF heartbeat detection, both classification performance and localization accuracy are equally important. In the AF-DETR model, 2D bounding boxes composed of center points and box widths are introduced to derive positional queries. The positional queries are encoded using sine positional encoding, similar to the positional encoding used for encoder features, ensuring the similarity between the position queries and the positional information in the encoder features. Deriving queries using coordinates enables the iterative updating of bounding boxes, and the progressively updated bounding boxes allow the construction of auxiliary losses using outputs from each decoding layer. These strategies accelerate model convergence and enhance both localization and classification accuracy.

Due to the stochastic nature of the training process, slight changes in the cost matrix can lead to significant variations in matching results, resulting in unstable bipartite matching and unstable model training. Denoise training can reduce the instability of bipartite matching to accelerate model convergence. In fact, denoise training bypasses bipartite matching because the correspondence between the noise query prediction and the truth value is known. By training the model to reconstruct boxes from noised boxes close to ground truth, denoise training allows the model to focus more on the nearby region of each query, preventing potential prediction conflicts between queries. Additionally, we construct positive and negative noise queries for the same ground truth object, which helps the model differentiate subtle differences between bounding boxes and avoids redundant predictions.

Considering factors that affect AF detection performance during model training is crucial, as it determines whether the trained model can generalize to unseen individuals. Employing appropriate strategies can enhance model performance. Firstly, the diversity of samples in the training set significantly impacts model performance. Typically, the number of participants in the dataset is directly related to sample diversity, as ECG samples from the same participant over a short period tend to be highly similar. Data augmentation methods are often used to enhance sample diversity. Secondly, while considering sample diversity, the balance of samples should not be overlooked. Imbalanced samples can reduce the model ability to recognize minority classes. However, its effects can be mitigated by sampling strategies or special loss functions. Additionally, differences between training and testing data in terms of lead configurations can lead to prediction failures. Therefore, in this work, we applied a polarity inversion (vertical flipping) augmentation to emulate physiological polarity differences between leads. As verified by the ablation in Section 4.3.3, the augmentation yields negligible impact on F-wave visibility while slightly improving cross-lead generalization. Future work will further incorporate lead-aware augmentations such as baseline wander, narrowband interference, and electrode noise to better reflect real-world ambulatory artifacts

Although the AF-DETR framework uses a fixed 400 ms heartbeat-box width, the additional ablation demonstrated that the performance is largely insensitive to moderate variations in box size. The adaptive-RR configuration provided only minimal gains, indicating that the empirical width adequately captures the P-QRS-T morphology under both bradycardic and tachycardic conditions. Therefore, the fixed-width design offers an effective trade-off between physiological coverage and computational simplicity.

Cross-database evaluation further confirmed that AF-DETR generalizes robustly across datasets with differing sampling rates and labeling styles, maintaining consistent performance within ±0.7% AF F1.

The additional analysis on MITDB demonstrated that premature atrial and ventricular ectopy are the dominant causes of false-positive AF detections. The use of focal loss or class-weighted cross-entropy significantly improved both precision and probability calibration, suggesting that ectopy-aware training objectives could further enhance model reliability in future studies.

The sensitivity and time-consecutive analyses verified that the 50% segment-level criterion approximates the clinical 30 s AF definition with high fidelity, confirming that the beat-level predictions of AF-DETR possess adequate temporal continuity for clinical interpretation.

### 5.3. Limitation and Future Work

Although there are many benefits, this study still has certain limitations. Firstly, the AF-DETR model did not consider the impact of different levels of noise on its performance. Another limitation is that there is still room for improvement in the positioning performance of the model, because the positioning performance affects the classification performance of the heartbeat. Lastly, the model still needs validation in more realistic clinical settings. In the future, we plan to validate our proposed AF-DETR method in clinical practice at hospitals, and expand datasets with diverse arrhythmia data collected from different environments for training and evaluation to achieve higher accuracy in AF heartbeat detection. Additionally, we aim to investigate its interpretability.

## 6. Conclusions

A novel AF object detection model called AF-DETR is proposed in this work, aiming to achieve localization and classification of AF heartbeats. The model adopts the DETR-like architecture and introduces a 2D bounding box in the transformer decoder to derive positional queries. Through the iterative bounding box refinement mechanism at each decoder layer, the model dynamically corrects prediction boxes, accelerating model convergence while simultaneously improving localization and classification performance. Additionally, contrastive denoising training is introduced to expedite model convergence and avoid redundant predictions for the same heartbeat. External independent testing results demonstrate that AF-DETR can achieve state-of-the-art performance in segment-level classification tasks, while providing accurate heartbeat-level classification labels and positions. Moreover, the localization performance of heartbeat detection is comparable to mainstream QRS detection algorithms. These results establish the effectiveness of the AF-DETR approach, enabling precise quantification of AF and providing valuable references for diagnosis by medical professionals.

## Figures and Tables

**Table 1:** Details of the datasets used in this study. Freq: Sampling frequency, NR: Total number of records, NS: Number of subjects in the recording, TD: Total duration, AFD: AF duration, NEB: Number of ectopic beats.

Dataset	Freq (Hz)	NR	NS	Lead	Record Length	Rhythms	TD	AFD	NEB
AFDB	250	25	25	ECG1, ECG2	10 h	4	234.28 h	93.40 h (39.87%)	N/A
CPSC2021	200	1436	105	I, II	0–6.8 h	3	480.19 h	164.44 h (34.24%)	93,545 (4.4%)
LTAFDB	128	84	84	ECG1, ECG2	6–26 h	9	1960.60 h	1030.89 h (52.58%)	285,100 (3.2%)
MITDB	360	48	47	II, V1,V2, V4, V5	0.5 h	15	24.07 h	2.21 h (9.18%)	34,442 (31.5%)
NSRDB	128	18	18	ECG1, ECG2	23–26 h	1	437.5 h	0 h (0%)	127 (0.007%)

**Table 2:** Data description after segmentation.

Dataset	Non-AF Segments	AF Segments	Non-AF Beats	AF Beats
AFDB	15,752	9461	586,321	455,171
CPSC2021	69,626	36,155	2,482,631	1,399,633
LTAFDB	100,067	115,755	3,510,824	5,023,006
MITDB	2413	220	90,132	9553
NSRDB	45,616	0	1,716,253	0

**Table 3:** The heartbeat positioning performance of AF-DETR on the external datasets.

Dataset	TP	FP	FN	Pre (%)	Sen (%)	MAE (ms)	MAE (Points)
AFDB	1,033,235	14,622	8257	98.60	99.21	13.71	1.75
LTAFDB	8,456,444	132,009	77,386	98.46	99.09	12.29	1.57
MITDB	99,231	1624	454	98.39	99.54	10.66	1.36
NSRDB	1,715,492	3676	761	99.79	99.96	5.81	0.74

**Table 4:** Beat-level classification performance on the external datasets.

	AF			Non-AF					
Dataset	Pre (%)	Sen (%)	F1 (%)	Pre (%)	Sen (%)	F1 (%)	Pre (%)	Sen (%)	F1 (%)
AFDB	96.23	95.35	95.79	96.88	98.62	97.74	96.56	96.99	96.77
LTAFDB	95.63	96.36	96.00	96.18	96.63	96.41	95.91	96.50	96.20
MITDB	73.02	98.76	83.96	98.35	95.96	97.14	85.69	97.36	90.55
NSRDB	/	/	/	99.79	99.94	99.87	/	/	/

**Table 5:** Segment-level classification performance on the external datasets.

		AF			Non-AF					
Dataset	Pre (%)	Sen (%)	F1 (%)	Pre (%)	Sen (%)	F1 (%)	Acc (%)	Pre (%)	Sen (%)	F1 (%)
AFDB	98.55	96.82	97.67	98.11	99.14	98.62	98.27	98.33	97.98	98.15
LTAFDB	97.58	97.86	97.72	97.52	97.20	97.36	97.55	97.55	97.53	97.54
MITDB	75.60	100	86.11	100	97.06	98.51	97.30	87.80	98.53	92.31
NSRDB	/	/	/	100	99.99	99.99	99.99	/	/	/

**Table 6:** Effect of the AF-beat ratio threshold (θ) on segment-level performance (CPSC2021→AFDB).

Threshold θ (AF-Beat Ratio)	Segment Acc (%)	AF F1 (%)	Non-AF F1 (%)
0.3	98.12	97.68	98.43
0.4	98.21	98.01	98.32
0.5 (default)	98.27	98.15	98.33
0.6	98.25	97.94	98.30
0.7	98.11	97.60	98.21

**Table 7:** Comparison between majority-vote and time-consecutive decision rules (CPSC2021→AFDB).

Decision Rule	Segment Acc (%)	AF Precision (%)	AF Recall (%)	AF F1 (%)
Majority-vote (>50%)	98.27	98.55	96.82	97.67
Time-consecutive (≥30 s continuous AF)	98.22	98.46	96.74	97.59

**Table 8:** Ablation study performance of AF-DETR on the MITDB dataset.

	Positioning			Beat-Level Classification		
Method	Pre (%)	Sen (%)	MAE (ms)	Pre (%)	Sen (%)	F1 (%)
AF-DETR	98.39	99.54	10.66	85.69	97.36	90.55
DN without CDN	98.79	99.85	6.25	81.67	96.85	87.39
No DN	95.80	97.61	19.26	83.86	94.27	88.33
No auxiliary loss	92.16	93.21	27.83	75.44	86.65	79.85

**Table 9:** Effect of bounding-box width on localization and AF classification performance.

Box Width Strategy	IoU	MAE (ms)	AF F1 (%)
Fixed 300 ms	0.921	15.84	97.68
Fixed 400 ms (default)	0.934	13.71	98.26
Fixed 500 ms	0.932	14.32	98.19
Adaptive RR (0.5 × R–R)	0.936	13.45	98.33

**Table 10:** Effect of polarity inversion augmentation on model performance.

Augmentation Strategy	IoU	MAE (ms)	AF F1 (%)
Without vertical flip	0.933	13.76	98.21
With vertical flip (default)	0.934	13.71	98.26

**Table 11:** Lead-swap robustness test (CPSC2021→AFDB).

Lead Configuration	IoU	MAE (ms)	AF F1 (%)
Train II→Test II (default)	0.934	13.71	98.26
Train I→Test II (lead-swap)	0.931	13.89	97.90

**Table 12:** Cross-database generalization performance (mean ± 95% CI over 3 runs).

Train Dataset→Test Dataset	IoU	MAE (ms)	AF F1 (%)
CPSC2021→AFDB (default)	0.934 ± 0.002	13.7 ± 0.3	98.26 ± 0.06
CPSC2021→MITDB	0.927 ± 0.003	14.1 ± 0.4	97.93 ± 0.08
AFDB→CPSC2021	0.923 ± 0.004	14.5 ± 0.5	97.61 ± 0.10
LTAFDB→AFDB	0.925 ± 0.003	14.3 ± 0.4	97.75 ± 0.09
CPSC + AFDB joint training→MITDB	0.936 ± 0.002	13.6 ± 0.3	98.34 ± 0.05

**Table 13:** Summarizes the rhythm-wise distribution of false-positive (AF) detections.

Confounding Rhythm	False Positives (Count)	Proportion of Total (%)
PAC (premature atrial contraction)	148	32.2
PVC (premature ventricular contraction)	121	26.3
Bigeminy (atrial/ventricular)	84	18.2
AV block (conduction delay)	39	8.5
Noise or motion artifact	68	14.8
Total	460	100.0

**Table 14:** The results for beat-level AF detection on MITDB under the different loss functions.

Loss Function	AF Precision (%)	AF Recall (%)	AF F1 (%)	Overall F1 (%)
Cross-entropy (default)	73.0 ± 0.3	98.7 ± 0.2	84.0 ± 0.3	90.6 ± 0.2
Focal loss (γ = 2, α = 0.75)	78.5 ± 0.4	98.0 ± 0.2	87.1 ± 0.3	92.1 ± 0.2
Class-weighted cross-entropy	76.3 ± 0.4	98.2 ± 0.3	86.1 ± 0.3	91.6 ± 0.2

**Table 15:** Comparison of the positioning performance between the proposed method and previous works.

Study	TP	FP	FN	Pre (%)	Sen (%)
Pan et al. [57]	97,794	2036	1891	97.96	98.10
Hamilton et al. [58]	94,075	8883	5610	91.37	94.37
Zong et al. [59]	99,043	6732	642	93.64	99.36
Christov et al. [60]	98,027	2894	1658	97.13	98.34
AF-DETR	99,231	1624	454	98.39	99.54

**Table 16:** Comparison of the classification performance between the proposed method and previous works.

Study	Year	Training Set	Test Set	Acc (%)	Pre (%)	Sen (%)	F1 (%)
Andersen et al. [33]	2019	AFDB	MITDB	87.40	45.45	98.96	/
		AFDB	NSRDB	95.01	/	/	/
Shi et al. [61]	2020	AFDB	MITDB	87.4	81.11	97.46	/
Seo et al. [62]	2021	AFDB	MITDB	86.68	/	/	/
Liu et al. [34]	2022	AFDB	MITDB	92.23	53.92	95.17	68.84
			NSRDB	96.86	/	/	/
Yun et al. [63]	2023	CPSC2021	LTAFDB	/	96.45	94.84	95.64
AF-DETR	2024	CPSC2021	LTAFDB	97.55	97.55	97.53	97.54
		CPSC2021	AFDB	98.27	98.33	97.98	98.15
		AFDB	MITDB	92.97	77.15	95.96	83.16
		AFDB	NSRDB	96.12	/	/	/

## Data Availability

The CPSC2021, AFDB, LTAFDB, and MITDB datasets are publicly available at https://physionet.org/content/cpsc2021/1.0.0/ (accessed on 7 October 2025), https://physionet.org/content/afdb/1.0.0/ (accessed on 7 October 2025), https://physionet.org/content/ltafdb/1.0.0/ (accessed on 7 October 2025) and https://physionet.org/content/mitdb/1.0.0/ (accessed on 7 October 2025), respectively.
